# The Automatic Design of Multimode Resonator Topology with Evolutionary Algorithms

**DOI:** 10.3390/s22051961

**Published:** 2022-03-02

**Authors:** Vladimir V. Stanovov, Sergey A. Khodenkov, Aleksey M. Popov, Lev A. Kazakovtsev

**Affiliations:** 1 Institute of Informatics and Telecommunications, Reshetnev Siberian State University of Science and Technology, 31 Krasnoyarsky Rabochy av., 660037 Krasnoyarsk, Russia; vladimirstanovov@yandex.ru (V.V.S.); hodenkov@sibsau.ru (S.A.K.); vm_popov@sibsau.ru (A.M.P.); 2 Institute of Space and Information Technologies, Siberian Federal University, 79 Svobodny pr., 660041 Krasnoyarsk, Russia; 3 Institute of Business Process Management, Siberian Federal University, 79 Svobodny pr., 660041 Krasnoyarsk, Russia

**Keywords:** multimode resonator, amplitude-frequency characteristics, microwave sensor, optimization, differential evolution, parameter adaptation

## Abstract

Microwave electromagnetic devices have been used for many applications in tropospheric communication, navigation, radar systems, and measurement. The development of the signal preprocessing units including frequency-selective devices (bandpass filters) determines the reliability and usability of such systems. In wireless sensor network nodes, filters with microstrip resonators are widely used to improve the out-of-band suppression and frequency selectivity. Filters based on multimode microstrip resonators have an order that determines their frequency-selective properties, which is a multiple of the number of resonators. That enables us to reduce the size of systems without deteriorating their selective properties. Various microstrip multimode resonator topologies can be used for both filters and microwave sensors, however, the quality criteria for them may differ. The development of every resonator topology is time consuming. We propose a technique for the automatic generation of the resonator topology with required frequency characteristics based on the use of evolutionary algorithms. The topology is encoded into a set of real valued parameters, which are varied to achieve the desired features. The differential evolution algorithm and the genetic algorithm with simulated binary crossover and polynomial mutation are applied to solve the formulated problem using the dynamic penalties method. The experimental results show that our technique enables us to find microstrip resonator topologies with desired amplitude-frequency characteristics automatically, and manufactured devices demonstrate characteristics very close to the results of the algorithm. The proposed algorithmic approach may be used for automatically exploring the new perspective topologies of resonators used in microwave filters, radar antennas or sensors, in accordance with the defined criteria and constraints.

## 1. Introduction

Microstrip resonators are traditionally used to build on their basis high-frequency frequency-selective devices with high-quality electrical characteristics, which are distinguished by manufacturability and low cost in mass production [1]. In addition, they are used in microstrip devices for measuring the electrical characteristics of various materials and substances, that is, they can be used as microwave sensor designs [2,3,4].

The rapid development of telecommunication technologies for the needs of tropospheric and space communications, radar and navigation systems [5], unmanned vehicles [2] is accompanied by a constant increase in the frequency-selective properties of microwave devices, such as bandpass filters, high and low pass filters, diplexers [6,7].

Such microwave bandpass filters [5,8,9,10] are also traditionally used in radio equipment. However, the development of these promising designs implies not only a constant improvement in their electrical characteristics and a reduction in size, but also a significant expansion of the operating bandwidth, which, accordingly, can significantly expand information transmission channels, or, on the contrary, cut off interference in a wide range. Therefore, the study of multimode microstrip resonators is an urgent and important task. Using the design features of a microstrip conductor, it is possible to significantly bring together several of its lowest oscillation modes in frequencies, which will form a wide bandwidth. It is important that, in terms of such parameters as miniaturization, reliability, manufacturability, and cost, devices based on such resonators [11], including ultra-wideband (UWB) ones, are among the best.

On the basis of microstrip resonators, it is possible to effectively implement both bandpass filters and notch filters (stop band filters) [12,13]. At the same time, the scope of microstrip resonators is not limited to frequency-selective designs. Microwave sensors designed on their basis are of high interest [14,15,16]. The concept of microwave sensors based on microstrip resonators is based on changes in their frequency response depending on the dielectric permittivity of the samples under test which are considered as an additional dielectric layer [14,17,18,19,20,21] or are introduced in the cleft of the proposed sensor [16,22].

Microwave sensors based on microstrip resonators [23] are used for measuring complex permittivity of materials, dielectric measurements of solids and liquids [24,25,26], gas sensing [15], non-invasive medical measurements [26,27,28,29] and other applications [30,31,32]. Permittivirt measurement methods of dielectric materials available in sheet, liquid, paste, powder, etc., forms by microwave resonators used as sensors were summarized in [33,34].

Moreover, the rapid development of the Internet of Things and sensor networks requires many miniature devices to work together in a limited frequency range without mutual interference [35]. Microstrip filter is an important component of wireless sensor network nodes, which selects useful signals and suppresses clutter interference signals. Multi-mode microstrip filters are required to prevent signal crosstalk between adjacent channels [36]. This requires high frequency selectivity and out-of-band rejection of the filters.

Particularly noteworthy are the designs of bandpass filters based on two-mode [1,22], three-mode [37] and multimode resonators. In the formation of the passband of such devices, in contrast to one-mode microstrip filters, the resonances of multiple lowest oscillation modes from each resonator are involved. As a result, the order of the filter multiplies, which is known to determine its selectivity. Due to the small number of links in such filters, not only the overall dimensions are significantly reduced in comparison with traditional designs, but the losses in the passband are also reduced. Strip conductors of multi-mode resonators in a microstrip filter are usually in the form of a square, a square or rectangular frame, sometimes they are made in a form of a closed meander line, which also has a fourth-order symmetry axis [5]. The natural frequencies of the first two oscillation modes in such resonators are degenerate, and the spatial distributions of the amplitudes of high-frequency fields are orthogonal. In this case, in a bandpass filter, the required amount of coupling between the resonances of two oscillation modes is provided either by etching one of the corners of a conductor in the shape of a square or square frame, or by adding a segment of a slotted line or a strip coupling element, which, as a rule, are located at an angle of 45° to the resonator axes [1].

Two-mode resonators with a regular strip conductor partially split at one end by a narrow slit [38] are more miniature. In this case, the value of the link between the first two modes of resonator oscillations is small, and it is implemented by a small difference in the length of the conductors in its split section. This circumstance does not allow us to develop the broadband filters based on such resonators; however, filters with a narrow passband on them have sufficiently high frequency-selective properties even in designs consisting of only two or three resonators [39].

Such frequency-selective devices allow us to implement them in multiple operation modes when a certain form of their strip conductors is set. As a result, the natural frequencies of several lowest oscillation modes could be brought together and miniaturize the structure [40].

Requirements for resonators may differ. In some cases (filters, radar antennas, etc.), the topology is required to cover a wide range of frequencies. At the same time, the prospects for designing sensor elements based on multimode microstrip resonators may be due to the ability to cover a wide range of interference when measuring the dielectric properties of substances. Specific types of sensors (e.g., gas sensors) require the use of a resonator with a very narrow bandwidth. Nevertheless, the problem of searching for a resonator design with a given center frequency, as well as with a given bandwidth, considered in this paper, is relevant for most types of devices

Due to their small circuit sizes (up to several millimeters in each dimension), such resonators can be used in hybrid integration with small wireless networking and radar devices [41,42]. Ultra-wideband (multi-mode) microwave filters are an essential part of the radar sensor systems [4,43,44] and antenna sensors [2] which are of high interest due to the development of unmanned vehicles and robotics. Such systems include both microstrip resonators with the simplest geometric configuration [45,46,47] and more complex designs [48,49].

For the sensor network nodes, it is essential that the central frequency points of the filter has a certain capacity bandwidth [35,50].

We consider the problem of computer-aided design of microstrip structures of multi-mode resonators with the strip conductors in the form of a combination of rectangles [1,37]. Filters based on such multi-mode resonators are implemented not only on substrates with a high dielectric constant, but also with a low one [1]. Such designs, due to the attenuation poles existing near the passband, are distinguished by an increased steepness of the amplitude-frequency characteristic (AFC) slopes, wide barrage bands and a high level of microwave power suppression in them which is essential for their application in sensor network nodes.

Well-known standard mathematical optimization methods such as the random search method, the Davidon-Fletcher-Powell algorithm, the similex Nelder-Mead method and genetic algorithms are implemented as subprograms in all popular software products for the design of microwave devices. An experienced user of these products can successfully optimize single mode resonator filters. Some promising results have been achieved in the development of methods for optimizing the designs of two-mode resonators [51]. Since it was stated that standard methods of mathematical programming were unsuitable even for the optimization of two-mode constructions, for the development of such devices, special optimization methods based on taking into account the physical principles and features of each specific design were proposed, thus not being in any way universal. In the computer-aided development of such systems, expert systems are widely used.

Modern software products designed for the numeric electrodynamic analysis of microwave device structures make it possible to simulate the amplitude-frequency response of a microstrip resonators of various types with high accuracy. The capabilities of modern computing systems enables us to perform electrodynamic analysis many times, while changing the geometric parameters of the microwave device. Electrodynamic analysis software products provide an application programming interface through which an external program can set the required device configuration.

The concept of this work is to develop evolutionary methods for optimizing the designs of microwave microstrip resonators including multimode ones, in which the well-known methods of electrodynamic analysis, implemented in modern software products, are used to assess the AFC, which, in turn, is used to calculate the fitness function in the evolutionary algorithms.

The recent advances in the area of optimization methods that are capable of solving a variety of problems, such as evolutionary algorithms (EAs) [52], open new possibilities for automation of many routines, including those present in the area of microwave filters design. In particular, the existing EAs, such as genetic algorithms (GAs) [53], particle swarm optimization (PSO) [54], differential evolution (DE) [55] and many others [56] do not require specific information about the type of the problem to be solved or information about the derivative of the fitness function. Nevertheless, when combined with certain constraint handling techniques, they enable us to efficiently solve many problems, which arise in industrial applications [57].

This study is focused on the application of one of the variants of DE, namely the L-SHADE algorithm [58] with several modifications, including mutation strategy, crossover operators and constraint handling technique, to the problem of designing the microstrip resonator topology. For this purpose, we propose an encoding method, allowing different topologies to be tested, and the specific settings for the optimization algorithm to be set.

The rest of the paper is organized as follows. The second section describes the methods used in our research and includes a description microstrip microwave resonators and their properties, description of the existing DE algorithm version and other real-coded evolutionary algorithms, and the proposed approach to the automated microwave device design. The experimental setup and results are demonstrated in the third section, and the last section concludes this study.

## 2. Materials and Methods

### 2.1. Microstrip Microwave Resonators

When calculating the amplitude-frequency characteristics (AFC) of all the considered resonators, we used monolithic substrates made of materials widely used in microwave technology (TBNS and Policor) with the same plate thickness h=1 mm but different relative permittivity ε = 80 and ε = 9.8, respectively.

The microwave resonators are characterized by certain criteria, which are functionals of the AFC. In this study, we propose an approach to the automated design of the resonators and implement it for three types of devices, namely the U-shaped, U-shaped with an additional cut and W-shaped resonators. The strip conductors of the resonators have the following geometric shapes: slot-split rectangular (U-shaped), twice slot-split rectangular (U-shaped with an additional cut), and studs with a plume (W-shaped), respectively. However, our approach does not depend on the shape of a flat microstrip resonator and can be implemented to the resonators of various shapes.

**Figure 1:**
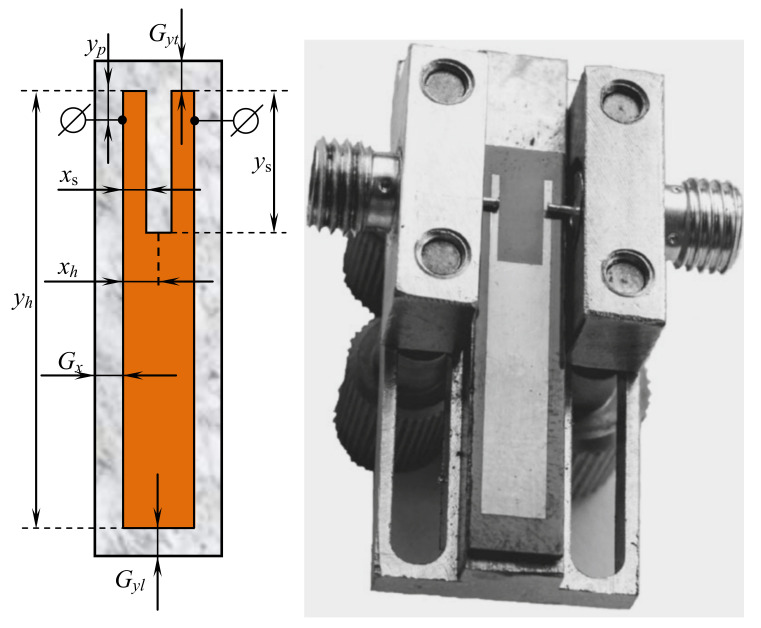
U-shaped (slot-split rectangular) 3-mode resonator: layout and manufactured device.

**Figure 2:**
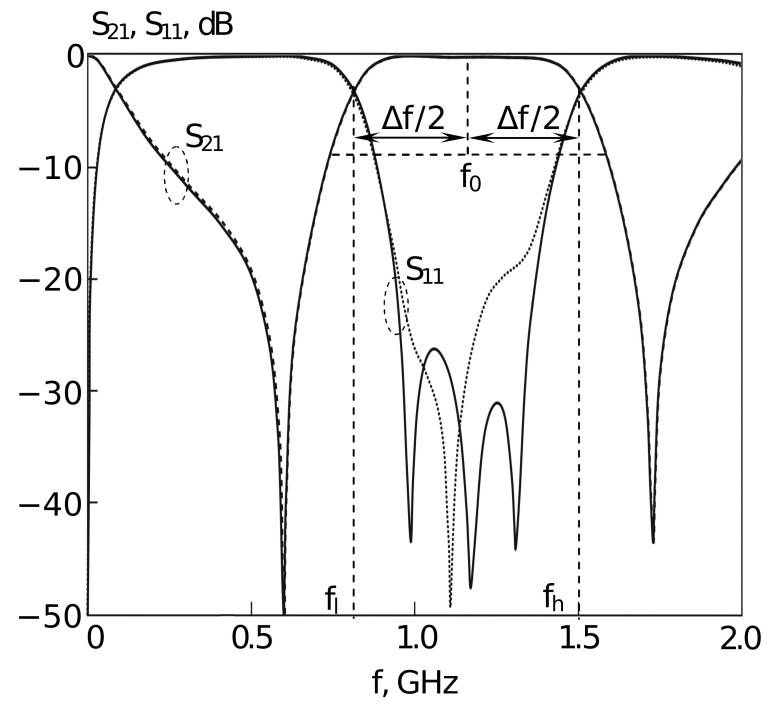
AFC of the U-shaped 3-mode (slot-split rectangular) resonator. Points—experimental data, lines—theoretical data (results of electrodynamic modelling).

The bandwidth of the first considered resonator type (Figure 1) can be formed both by its two lowest oscillation modes [38] and by three ones (Figure 2) [39]. Changing the width and depth of the cut in the conductor makes it possible to bring these lower natural frequencies closer together in such a resonator. In this case, the relative bandwidth $dF=\Delta F/F_{0}$ of such a two-mode resonator does not exceed 40%, where ΔF is the bandwidth measured at the level of −3 dB from the level of the minimum loss in the bandwidth, $F_{0}$ is the center frequency of the bandwidth. For the design of devices with a wide frequency range, a three-mode resonator is more promising. Our studies using Policor dielectric plates, at a fixed central frequency of the resonator $F_{0}=3$ GHz show that its minimum bandwidth is 45%, and the maximum bandwidh is 71%. The first resonator was set to operate in a three-resonance mode [39], its structure is shown in Figure 1. Such a U-shaped resonator is a very simple construction which is one of the most widely used in various microwave devices including sensors [3,59,60,61,62]. Depending on the dimensions, such devices have various AFCs and purposes. Similar forms such as Y-shaped resonators are also popular [63]. The theoretically calculated data for a three-mode resonator implemented on a TBNS dielectric plate, obtained using a numerical electrodynamic analysis of its 3D model, are in almost ideal agreement with the data taken on an actually manufactured prototype [39].

When splitting the strip conductor of the MPR by a narrow slit [38], it is possible to bring the natural frequencies of its first two oscillation modes closer together. This allows such two-mode resonators to create not only band-pass filters with high frequency-selective properties, but also two-band filters, as well as diplexers. However, the relative bandwidth of such filters does not exceed 20%, while the investigated resonators make it possible to develop filters with bandwidths up to 70%, and such devices have high frequency-selective properties.

In addition, Figure 1 shows the main geometrical parameters required to encode such a resonator for the optimization algorithm. The top and the lower gaps are $G_{yt}$ and $G_{yl}$, respectively, and the horizontal gap is $G_{x}$, which is the same on both sides. Mentioned gaps are fixed during experiments: if the resonator size changes, then the enclosure size also changes to keep the same gaps. The resonator has the following geometrical properties:

1. $x_{h}$—half of resonator width, in mm (only one side is encoded and then mirrored);
2. $y_{h}$—resonat’s height, in mm;
3. $x_{s}$—relative width of the upper part, $x_{s}\in \left[0,1\right]$, i.e., if $x_{s}=1$ then the width is equal to $x_{h}$;
4. $y_{s}$—relative height of the upper part, $y_{s}\in \left[0,1\right]$, i.e., if $y_{s}=0$ then the height is equal to 0 (no central cut);
5. $y_{p}$—relative height of the position of the side conductors to ports, $y_{p}\in \left[0,1\right]$, i.e., if $y_{p}=0$, then the conductors are located at the top.

The following parameters of the resonator are shown in Figure 1: $G_{x}=35$ mm, $G_{yl}=20$ mm, $G_{yt}=28$ mm, $x_{h}=13.25$ mm, $y_{h}=174.16$ mm, $x_{s}=0.61$, $y_{s}=0.325$, $y_{p}=0.019$. Depending on the ratio of these parameters, devices with completely different amplitude-frequency characteristics and purposes can be obtained [3,60,62]. Figure 2 shows the results of the AFC measurement performed using the electrodynamic numerical analysis for the resonator presented in Figure 1.

Figure 2 shows the frequency dependence of the forward loss for the passage of microwave power (transmission factor S21), as well as the frequency dependence of the reflection loss of microwave power (transmission factor S11). The most important parameters that characterize the resonator are the bandwidth $\Delta F=F_{h}-F_{l}$, where $F_{h}$ and $F_{l}$ are the high-frequency and low-frequency boundaries of the bandwidth measured at the level of −3 dB from the level of minimum losses in the bandwidth, and also the center frequency of the passband $F_{0}$. For the resonator in Figure 1, these parameters were as follows: $F_{0}=1.2$ GHz. Therelative bandwidth dF, measured as $dF=\frac{F_{h}-F_{l}}{F_{0}}$ is more convenient for the use in optimization algorithm than ΔF. In our case, dF=0.7. Both $F_{0}$ and dF are functionals of the AFC.

**Figure 3:**
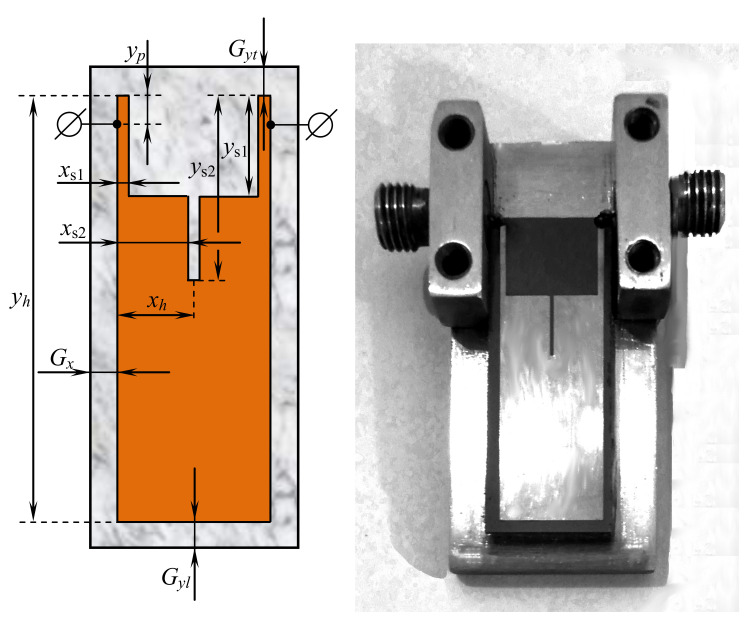
U-shaped (twice slot-split rectangular) 6-mode resonator with an additional cut: layout and manufactured device.

**Figure 4:**
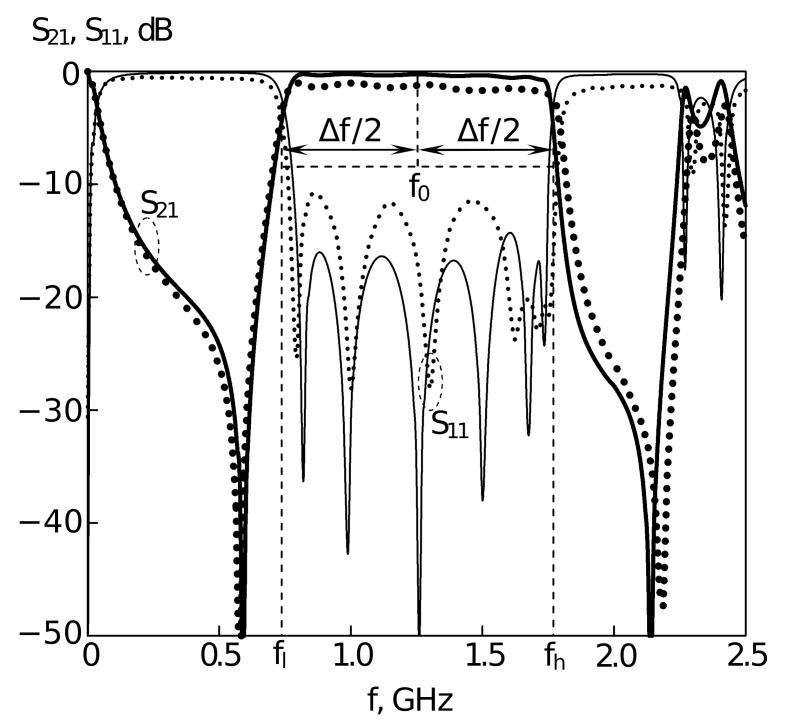
AFC of the U-shaped (twice slot-split rectangular) 6-mode resonator with an additional cut. Points – experimental data, lines – theoretical data (results of electrodynamic modelling).

Figure 3 shows the topology of the 6-mode microstrip resonator with a doubly slotted strip conductor. All theoretical and experimental studies for this resonator were carried out using a substrate with a relative permittivity ε=9.8 (Polycor). In this case, just as for a three-mode resonator implemented on a substrate with a high dielectric constant, the data for a six-mode resonator (Figuse Figure 4) obtained by numerical electrodynamic analysis of its 3D model are in good agreement with the data taken on a really manufactured prototype. This allows us to confine ourselves in further studies of microstrip resonators to only numerical experiments, and, moreover, conduct such experiments in an automated mode under control of an optimization algorithm.

At a fixed center frequency $F_{0}=3$ GHz, its minimum bandwidth is 79%, and the maximum one is 81%, which is significantly wider than that of a three-mode resonator.

The 6-mode resonator, considered in this study, is similar to the 3-mode one, however, it has an additional cut in the middle. The parameters used to describe this resonator are similar to those of the previous one:

1. $x_{h}$—half of resonator width, in mm (only one side is encoded and then mirrored);
2. $y_{h}$—resonator height, in mm;
3. $x_{s1}$—relative width of the upper part, $x_{s1}\in \left[0,1\right]$, i.e., if $x_{s1}=1$ then the width is equal to $x_{h}$;
4. $y_{s1}$—relative height of the upper part, $y_{s1}\in \left[0,1\right]$, i.e., if $y_{s1}=0$ then the height is equal to 0 (no central cut);
5. $x_{s2}$—relative width of the small central cut, $x_{s2}\in \left[0,1\right]$, i.e., if $x_{s2}=1$ then the width is equal to $x_{h}$;
6. $y_{s2}$—relative height of the small central cut, $y_{s2}\in \left[0,1\right]$, i.e., if $y_{s2}=0$ then the height is equal to 0 (no central cut);
7. $y_{p}$—relative height of the position of the side conductors to ports, $y_{p}\in \left[0,1\right]$, i.e., if $y_{p}=0$, then the conductors are located at the top.

The following parameters of the resonator are shown in Figure 3: $G_{x}=35$ mm, $G_{yl}=24$ mm, $G_{yt}=30$ mm, $x_{h}=49.5$ mm, $y_{h}=280$ mm, $x_{s1}=0.121$, $y_{s1}=0.236$, $x_{s2}=0.969$, $y_{s2}=0.204$, $y_{p}=0.02$. Figure 4 shows the results of the AFC measurement performed using the electrodynamic numerical analysis for the resonator presented in Figure 3. The study was carried out using a substrate with a relative permittivity ε=9.8.

In Figure 4, the frequency dependence of direct losses from port 1 to port 2 and frequency dependence of losses in reflection, from port 1 to port 1, are shown. For this case, $F_{0}$ is around 1.26 GHz, and dF=0.8.

**Figure 5:**
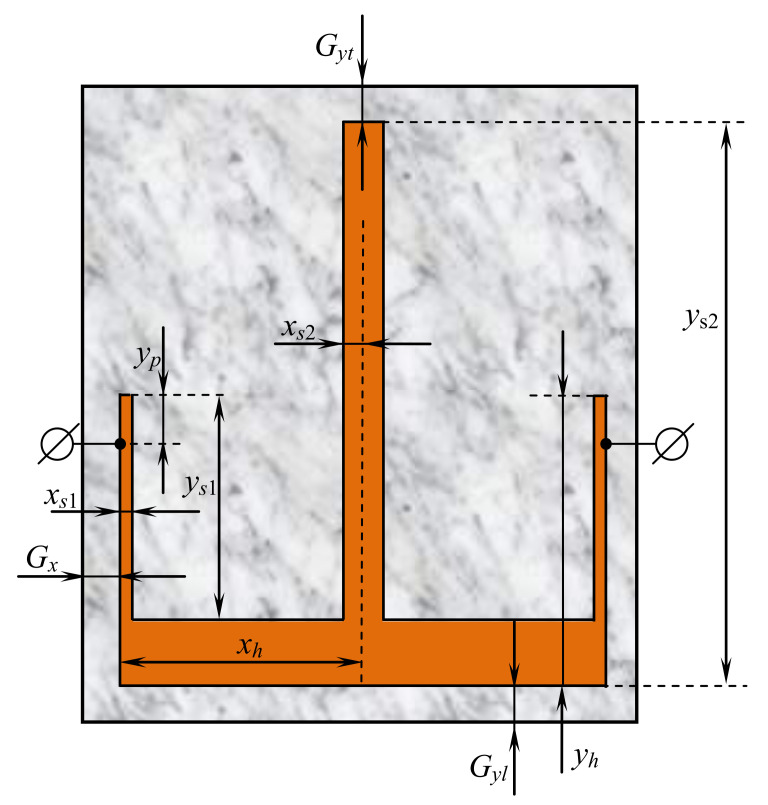
W-shaped (studs with a plume) 5-mode resonator: layout.

Figure 5 shows the topology of the 5-mode W-shaped (studs with a plume) microstrip resonator. Variation in the length of the stripe segment of the plume is accompanied by a decrease in the frequencies of the even modes of oscillation of this resonator, while the frequencies of its odd modes remain unchanged. This makes it possible to form the passband of this resonator by its five lowest resonances, while the relative width of its passband is 91%. We point out that dF can be varied from 74% to 97%, which allows us to recognize it as the most promising for building ultra-wideband devices, among the resonators considered in this paper.

The 5-mode resonator considered in this study has a W-shaped topology. The following parameters are used to describe this resonator:

1. $x_{h}$—half of resonator width, in mm (only one side is encoded and then mirrored);
2. $y_{h}$—resonator height (without central part), in mm;
3. $x_{s1}$—relative width of the side part, $x_{s1}\in \left[0,1\right]$, i.e., if $x_{s1}=1$ then the width is equal to $x_{h}$;
4. $y_{s1}$—relative height of the side part, $y_{s1}\in \left[0,1\right]$, i.e., if $y_{s1}=0$ then the height is equal to 0;
5. $x_{s2}$—relative width of the small central cut, $x_{s2}\in \left[0,1\right]$, i.e., if $x_{s2}=1$ then the width is equal to $x_{h}$;
6. $y_{s2}$—relative height of the small central cut, $y_{s2}\in \left[0,3\right]$, i.e., if $y_{s2}=0$ then the height is equal to 0 (no central cut);
7. $y_{p}$—relative height of the position of the side conductors to ports, $y_{p}\in \left[0,1\right]$, i.e., if $y_{p}=0$, then the conductors are located at the top.

**Figure 6:**
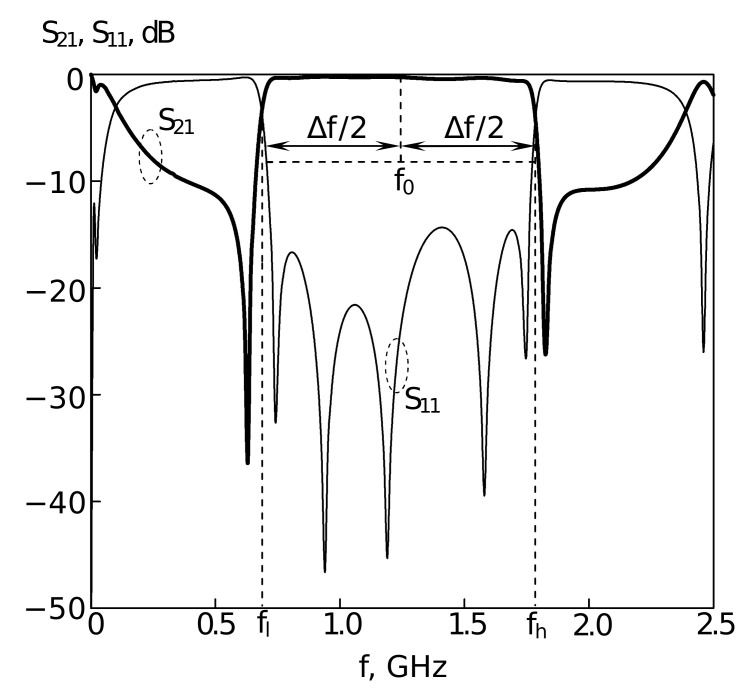
AFC of the W-shaped (studs with a plume) 5-mode resonator. Points – experimental data, lines – theoretical data (results of electrodynamic modelling).

Thus, the following parameters of the resonator are demonstrated Figure 5: $G_{x}=35$ mm, $G_{yl}=24.5$ mm, $G_{yt}=30$ mm, $x_{h}=78$ mm, $y_{h}=93.5$ mm, $x_{s1}=0.0384$, $y_{s1}=0.770$, $x_{s2}=0.075$, $y_{s2}=1.98$, $y_{p}=0.0145$. Figure 6 shows the results of the AFC measurement performed with use of electrodynamic numerical analysis for the resonator presented in Figure 5.

The described microstrip resonators will be further considered in this study for tuning their passband frequency by changing the topology parameters. The search of optimal resonator topology will be performed with the purpose of tuning the known topology to work in a different frequency ranges: for example, the resonator shown in Figure 3 has $F_{0}=1.26$ GHz with dF=0.8, and the goal will be to increase the frequency to 1.5 GHz with the same relative bandwidth dF=0.8. A simple scaling of the resonator geometrical properties does not give a proper result in most cases: the resulting frequency range is different from the desired as well as the width ΔF. Moreover, the number of modes could be changed.

### 2.2. Evolutionary Continuous Optimization Methods

Among the existing optimization methods for continuous search spaces, the evolutionary algorithms are considered as the most efficient global optimization techniques, as long as they are capable of finding global optimum even for complicated landscapes due to their population-based structure and randomized search. Various evolutionary algorithms are a popular and efficient tool for microelectronic device design [64,65,66] as well as data processing for evaluation of their quality [67,68]. Therefore, in this study, two evolutionary optimizers were considered: the differential evolution algorithm and the genetic algorithm (GA). The next two subsections provide a brief description of these methods.

### 2.3. Differential Evolution

The Differential Evolution is a population-based optimization method, originally proposed by Storn and Price in [69]. The main idea of the DE approach was to use the difference vectors between individuals in the population and apply these scaled vectors to the existing points in the search space to sample new solutions. DE appeared to be very efficient and easy to implement, although it has a small number of parameters. Such algorithms are of a high demand in various areas including sensor design [70]. The algorithm starts with a set of NP initial points in a *D*-dimensional search space, $x_{i,j}$, i=1…NP, j=1…D, generated using uniform distribution within $\left[xmin_{j},xmax_{j}\right]$, j=1…D. After initialization, the main loop starts, which includes mutation, crossover and selection.

There are several mutation strategies used in DE, with rand/1, used in [69], and current-to-pbest/1, proposed for the JADE [71] algorithm, being the most popular ones. The current-to-pbest/1 strategy is described with the following equation:

(1) $$v_{i,j}=x_{i,j}+F\cdot \left(x_{pbest,j}-x_{i,j}\right)+F\cdot \left(x_{r1,j}-x_{r2,j}\right),$$

where pbest is an index of one of the p·100% best individuals, r1 and r2 are randomly chosen indexes, and pbest≠r1≠r2≠i, *F* is the scaling factor parameter, usually in range [0,1], *v* is the set of mutant vectors, and *x* are called target vectors.

After the mutant vectors *v* are generated by mutation, the crossover is performed to generate trial vectors *u*. The most used method is the binomial crossover, in which the trial vector receives coordinates from mutant vector with probability Cr∈[0,1]. The binomial crossover could be described as follows:

(2) $$u_{i,j}=\left\{\begin{array}{cc} v_{i,j}, & \mathrm{if}\;rand(0,1)<Cr\;\mathrm{or}\;j=jrand\\ x_{i,j}, & \mathrm{otherwise} \end{array}\right.,$$

where jrand is a randomly generated index in [1,D]. It is needed to make sure that at least one component of the trial vector is different from the target vector. Otherwise, the trial vector would be exactly the same as target vector, which would lead to unnecessary calculations. After the set of trial vectors is generated, the bound constraint handling (BCH) method is applied. One of the widely used bound constraint handling methods [72] is described as follows:

(3) $$u_{i,j}=rand\left(xmin_{j},xmax_{j}\right)\;\mathrm{if}\;u_{i,j}\notin \left[xmin_{j},xmax_{j}\right].$$

Here, if the newly generated point is out of bounds, its parent is used to set the new position. However, in this study the following BCH approach was used:

(4) $$u_{i,j}=\left\{\begin{array}{cc} \frac{xmin_{j}+x_{i,j}}{2}, & \mathrm{if}\;u_{i,j}<xmin_{j}\\ \frac{xmax_{j}+x_{i,j}}{2}, & \mathrm{if}\;u_{i,j}>xmax_{j} \end{array}\right..$$

Here, $xmin_{j}$ and $xmax_{j}$ are the lower and the upper boundaries for the *j*-th variable. After the fitness values f(u) are calculated, the selection step is performed:

(5) $$x_{i}=\left\{\begin{array}{cc} u_{i}, & \mathrm{if}\;f\left(u_{i}\right)\leq f\left(x_{i}\right)\\ x_{i}, & \mathrm{if}\;f\left(u_{i}\right)>f\left(x_{i}\right) \end{array}\right..$$

If the newly generated solution $u_{i}$ is not worse than its parent individual $x_{i}$ from the population, then the replacement occurs. Such a selection operator is greedy, and it allows DE to solve multimodal problems efficiently.

### 2.4. L-SHADE Algorithm

One of the main problems of the DE algorithm is its sensitivity to three main parameters: scaling factor *F*, crossover rate Cr and population size NP. Therefore, in many studies, the parameter adaptation techniques have been proposed for DE, for example, the previously mentioned JADE algorithm [71]. Based on the JADE parameter adaptation, the SHADE [73] and L-SHADE [58] approaches were developed, which appeared to be highly competitive and popular nowadays, according to recent studies [74]. Below, the L-SHADE approach will be described.

The SHADE and the L-SHADE algorithms use the Success-History Adaptation (SHA) with a set of H=5 memory cells [73], each containing a pair of $M_{F,k}$ and $M_{Cr,k}$ values. These values are used to sample new parameters for *F* and Cr used in mutation and crossover, respectively. The sampling is performed by using the values in a randomly chosen historical cell with index k∈[1,H] in the following way:

(6) $$F=randc(M_{F,k},0.1),$$

(7) $$Cr=randn(M_{Cr,k},0.1),$$

where randc(m,s) is the Cauchy distributed random value and randn(m,s) is a normally distributed random value, with position and scale parameters *m* and *s* respectively. If the value generated for Cr is outside [0,1], it is truncated to this range. If the generated value F>1, then it is set to 1, however, in case if F<0, it is generated again, until it gets within the range [0,1], as F=0 means no mutation.

The successful values of *F* and Cr, i.e., those giving an improvement in terms of fitness value, are stored into $S_{F}$ and $S_{Cr}$ arrays, as well as the improvement values Δf=|f(u)−f(x)|. These values are used to update the memory cell with index *h*, which is incremented by 1 every generation, and when *h* exceeds memory size *H*, it is reset back to 1. The memory cells for both *F* and Cr are updated in a similar way with weighted Lehmer mean:

(8) $$mean_{wL}=\frac{\sum_{j=1}^{\left|S\right|}w_{j}S_{j}^{2}}{\sum_{j=1}^{\left|S\right|}w_{j}S_{j}},$$

where $w_{j}=\frac{\Delta f_{j}}{\sum_{k=1}^{\left|S\right|}\Delta f_{k}}$ and *S* is either $S_{Cr}$ or $S_{F}$. The previous values in the memory cells are replaced by new ones.

In addition to the success-history adaptation for *F* and Cr parameters, the L-SHADE algorithm uses the Linear Population Size Reduction (LPSR) technique. In this method the population size NP is initially set relatively large, and it is recalculated at the end of each generation. The new population size follows the linear function, depending on the available computational resource:

(9) $$NP_{g+1}=round\left(\frac{NP_{min}-NP_{max}}{NFE_{max}}NFE+NP_{max}\right),$$

where $NP_{min}=4$ and $NP_{max}$ are the minimal and initial population sizes, NFE and $NFE_{max}$ are the current and maximal number of function evaluations. At the end of each generation none, one or several worst individuals are removed from the population.

The standard L-SHADE algorithm also maintains an archive of inferior solutions *A*, which contains parent solutions replaced by better offspring. The archive is initially empty, and it is filled during the selection step: if an offspring is better than its parent, then the parent is stored in the archive. Once the archive reaches its maximum size NA, usually equal to the population size NP, its update scheme is changed: the parent individuals that should be put into the archive replace randomly chosen archive members. The archive size NA is reduced in the same manner as the population size NP.

### 2.5. Genetic Algorithm for Continuous Optimization

The Genetic Algorithms were originally proposed for binary or discrete optimization; however, there were multiple attempts to make them work in continuous search spaces. In one of the earliest studies [75], the Simulated Binary Crossover (SBX) was proposed: it imitates the behaviour of the single-point binary crossover operator. Among the existing crossover schemes the SBX has received significant attention and is widely used together with the polynomial mutation strategy [76]. Further on the description of a classical scheme of the GA approach with mentioned search operators will be given.

The continuous genetic algorithm starts with randomly initializing a set of NP solutions within the borders $\left[xmin_{j},xmax_{j}\right]$. After fitness calculation and remembering the best solution the main loop of the algorithm starts. The main loop contains the following steps:

1. selection of parents for crossover;
2. crossover of parents to generate offspring solutions;
3. mutation of the offspring solutions;
4. replacement of parents with the offspring.

There are several known selection schemes, such as proportional selection, rank-based selection and tournament selection. The last one is used more often due to its simplicity: a set of *T* individuals is randomly chosen from the population, and the individual having the best fitness value (winner of the tournament) becomes one of the parents. Typically, T=2 (binary tournament selection). For each crossover two parents $x^{1}$ and $x^{2}$ are chosen.

The SBX is performed on a pair of parents to produce offspring solutions using the following procedure [77]. First, a uniformly distributed random number *u* is generated in [0,1], next the $\beta_{q}$ value is calculated as follows:

(10) $$\beta_{q}=\left\{\begin{array}{cc} {\left(2u\right)}^{\frac{1}{\eta +1}}, & \mathrm{if}\;u<0.5\\ {\frac{1}{2(1-u)}}^{\frac{1}{\eta +1}}, & \mathrm{otherwise}\; \end{array}\right.,$$

where η is the non-negative crossover rate parameter that is responsible for the offspring spread, smaller η leads to larger spread. The value $\beta_{q}$ is used to calculate two offspring solutions:

(11) $$t_{j}^{1}=0.5\left[\left(1+\beta_{q}\right)x_{j}^{1}+\left(1-\beta_{q}\right)x_{j}^{2}\right],$$

(12) $$t_{j}^{2}=0.5\left[\left(1-\beta_{q}\right)x_{j}^{1}+\left(1+\beta_{q}\right)x_{j}^{2}\right],$$

where $t_{j}^{1}$ and $t_{j}^{2}$ are the offspring coordinates for variable j=1,2…D. The generated offspring solutions are symmetric about their parents, so no bias towards one of them is present. One of the offspring solutions is randomly chosen to be mutated and moved to the text generation.

Although the SBX generates solutions following a specified probability distribution, i.e., the offspring solutions are sampled around parents, the mutation step could be also used. The polynomial mutation [78] works in a similar way. First a random value *u* is sampled uniformly within [0,1]. Next, the $\delta_{q}$ parameter is calculated:

(13) $$\delta_{q}=\left\{\begin{array}{cc} {\left[2u+\left(1-2u\right){\left(1-\delta \right)}^{\left(\eta_{m}+1\right)}\right]}^{\frac{1}{\eta_{m}+1}}-1, & \mathrm{if}\;u<0.5,\\ 1-{\left[2\left(1-u\right)+2\left(u-0.5\right){\left(1-\delta \right)}^{\left(\eta_{m}+1\right)}\right]}^{\frac{1}{\eta_{m}+1}}, & \mathrm{otherwise}\;, \end{array}\right.$$

where $\delta =\frac{min(t_{j}-xmin_{j},xmax_{j}-t_{j})}{xmax_{j}-xmin_{j}}$ and $\eta_{m}$ is the non-negative distribution parameter (smaller values result in smaller dispersion). The mutated individual’s coordinate is calculated as follows:

(14) $$t_{j}=t_{j}+\delta_{q}\left(xmax_{j}-xmin_{j}\right).$$

The polynomial mutation is applied to each component of the offspring vector with a probability of 1/D. After the mutation, the replacement of parents with the offspring is performed. There are several well-known replacement schemes, for example:

1. offspring only;
2. offspring plus the copy of the best individual;
3. the best of offspring solutions and parents.

In the first case, all the offspring solutions *t* are copied to the parents *x* population regardless of their fitness values. This may lead to premature convergence and even loss of genetic diversity as well as losing the best known solution, if it is not stored separately. The second scheme makes sure that the best solution, either from the parents or the offspring, is copied to the new population. The third scheme is more conservative: it sorts the joined set of parents and offspring by fitness and selects the best NP solutions. This ensures that the best solution is always in the population, and if all the offspring solutions were unsuccessful, they will not be copied to the new population.

The described general scheme of GA is not the only one possible, and there are numerous known implementations of this algorithm, however, the given description is sufficient for this study.

### 2.6. Proposed Approach

As long as this study is focused on applying optimization methods to a specific real-world engineering problem, i.e., optimizing resonators topology, several optimization techniques were implemented. The algorithms proposed here are based on both GA and DE, described above, and have several modifications. First of all, there will be two search schemes used: based on differential evolution search operators and based on SBX and polynomial mutation. These two search schemes are based on DE and GA, although they do not fully follow the description given above. The main difference is the replacement mechanism, for which there will be three different scenarios considered, namely:

1. the best of the offspring and parents (replacement type RT=1);
2. random fitness-based replacement (replacement type RT=2);
3. pairwise comparison (DE selection, replacement type RT=3).

The first and third schemes have been already discussed. The second is performed as follows: after the best solution is saved to the population, for each offspring solution, a random parent index r1 is generated in [1,NP], and if the offspring is better than the r1-th parent, the replacement occurs. This replacement scheme combines the first and the third ones.

In the case if the GA approach is used, the linear population size reduction (LPSR) is applied in the same manner as in L-SHADE algorithm. Both η and $\eta_{m}$ parameters are fixed during the algorithm run. GA applies SBX and polynomial mutation to parents, selected by tournament selection with T=2. The crossover rate $CR_{g}$, i.e., the ratio of parent vectors components altered by SBX, was set to a constant value. The $CR_{g}$ was used to sample Cr values as the mean parameter in normal distribution with ς=0.1, i.e., the Cr sampling was similar to the one used in the L-SHADE algorithm. This algorithm will be further called L-CGA - Continuous Genetic Algorithm with LPSR (algorithm type AT=1).

In the case if the DE is used, the L-SHADE algorithm with current-to-pbest/1 mutation is applied, however, instead of the crossover operator the SBX from GA is applied for target vector $x_{i}$ and trial vectror $u_{i}$, i=1,…,NP. Next, the polynomial mutation is applied with a probability of $\frac{1}{D}$. The SHA is used to tune only *F* parameter, without tuning ηand $\eta_{m}$ for SBX and polynomial mutation. The crossover probability Cr was sampled using $CR_{g}$ parameter as described above. This algorithm will be further referred to as L-SHADGA: Success-History Adaptive Differential Genetic Algorithm with LPSR (algorithm type AT=2). In our algorithms, we use no archive (NA=0).

As it was mentioned above, the goal of the resonator topology optimization in this study is to perform tuning to the different frequency, while keeping the desired parameters. This is a constrained optimization problem, as it is required that the frequency dependence of losses in reflection should not exceed −14 dB in the area between $F_{l}$ and $F_{h}$. Moreover, the number of modes should be the same as defined: not smaller and larger than that of the basic topology. Thus, there should be 3 modes for the first resonator, 6 for the second and 5 for the third resonators respectively.

The constrained optimization problems could be solved with evolutionary algorithms in several ways. One of the most popular methods consists in the implementation of the penalty-based constraint handling techniques: the fitness function contains not only the difference between actual and desired frequency and bandwidth, but also a penalty on the number of modes with frequency dependence of losses exceeding −14 db in reflection. In this study the dynamic penalties method will be used to calculate fitness values as follows:

(15) $$Fit\left(x_{i}\right)=f\left(x_{i}\right)+g^{2}P\left(x_{i}\right),$$

(16) $$P\left(x_{i}\right)=\frac{pen(x_{i})}{5}PM^{2},$$

(17) $$f\left(x_{i}\right)=|F_{0}^{act}\left(x_{i}\right)-F_{0}^{des}|+100|dF^{act}\left(x_{i}\right)-dF^{des}|,$$

(18) $$pen\left(x_{i}\right)=pdb\left(x_{i}\right)(1+10|nmod^{act}\left(x_{i}\right)-nmod^{des}|)+50|nmod^{act}\left(x_{i}\right)-nmod^{des}|,$$

(19) $$pdb\left(x_{i}\right)=\left\{\begin{array}{cc} AFC_{1-1,[F_{l},F_{u}]}^{max}+14, & \mathrm{if}\;AFC_{1-1,[F_{l},F_{u}]}^{max}>-14\\ 0, & \mathrm{otherwise}\; \end{array}\right.,$$

where *g* is the current generation number, PM is the penalty magnitude parameter, $F_{0}^{des}$, $dF^{des}$ and $nmod^{des}$ are the desired values for frequency, bandwidth and number of modes respectively, $F_{0}^{act}\left(x_{i}\right)$, $dF^{act}\left(x_{i}\right)$ and $nmod^{act}\left(x_{i}\right)$ are the actual values for solution $x_{i}$.

**Figure 7:**
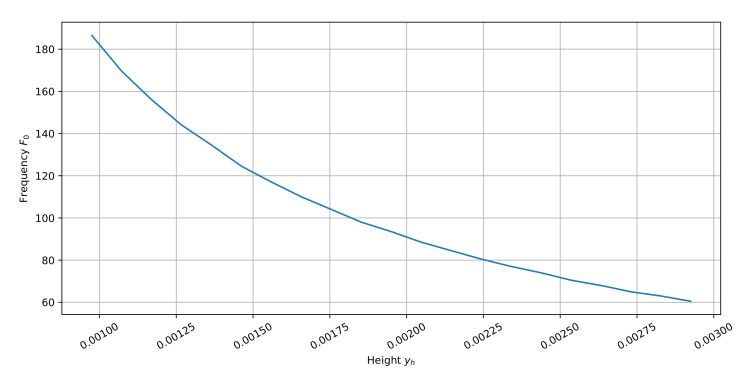
Dependence of $F_{0}^{act}$ on $y_{h}$ values.

Although the scaling of the resonator does not allow simple frequency tuning, it is possible to use the scaled solution as the baseline during initialization. To figure out the required scaling to tune the resonator to the area of desired frequency $F_{0}^{des}$, a set of measurements has been made for the 3-mode U-shaped resonator. The result of $F_{0}^{act}$ measurements for different $y_{h}$ values is shown in Figure 7.

Based on the observed dependence the following scaling procedure is proposed:

(20) $$x_{h}^{sc}=x_{h}^{bas}\frac{F_{0}^{bas}}{F_{0}^{goal}},$$

(21) $$y_{h}^{sc}=y_{h}^{bas}\frac{F_{0}^{bas}}{F_{0}^{goal}},$$

where $x_{h}^{bas}$ and $y_{h}^{bas}$ are baseline parameters of the given resonator, $x_{h}^{sc}$ and $y_{h}^{sc}$ are scaled values used during initialization, $F_{0}^{bas}$ is the frequency of the baseline topology. It should be noted that although in Figure 7 only the dependence on $y_{h}$ is shown, for all three resonators in this study $x_{h}$ does not have such significant influence, however, scaling it as well as $y_{h}$ enables us to find better solutions simpler.

The initialization is performed not uniformly, but close to the scaled solution. In particular, the first individual $x_{1}$ has the scaled $x_{h}$ and $y_{h}$ values, while the rest individuals are generated with normal distribution: $x_{i,j}=x_{1,j}*randn\left(1,0.02\left(xmax_{j}-xmin_{j}\right)\right)$, where randn(1,ς) is a normally distributed random value with mean equal to 1 and standard deviation ς. The parameter value is sampled until it falls within $\left[xmin_{j},xmax_{j}\right]$ interval. This initialization allows most of the population to be sampled near the scaled solution, resulting in sufficient diversity, allowing an efficient start of the search process.

In the next section, the details of the performed computational experiments are given, including parameter settings of the used algorithms as well as the final results, approaches comparison, and the applicability of the results for constructing various devices.

## 3. Results and Discussion

One of the most important problems with optimizing the microstrip resonator topology is the computational complexity of the fitness function: modeling the AFC of the resonator in a specified frequency range may take from 5 to 60 s. Resonators used as sensors may have very simply [15,16,45,46,79] or rather sophisticated topology [17,18,80]. For more complex topology, the modelling can take more time. Therefore, this should be considered as expensive optimization problem, where every evaluated solution is gained through significant amount of calculations.

The search algorithms were implemented in Matlab and ran on a Windows 10 system with 8-core AMD Ryzen 3700X processor.

One of the problems addressed during the investigation was the development of recommendations about the algorithmic schemes, which would be preferable for resonator topology optimization. In particular, the sensitivity analysis was performed for each of the three baseline resonator topologies. The sensitivity analysis included varying three main parameters for two search algorithms (L-CGA, AT=1 and L-SHADGA, AT=2) with three replacement schemes (RT=1,2,3), including:

1. penalty Magnitude (PM=1,2,3,4,5);
2. crossover and mutation distribution parameters ($\eta =\eta_{m}=15,30,45,60,75$);
3. crossover rate parameter ($CR_{g}=0.2,0.4,0.6,0.8,1.0$).

Not all the possible combinations were considered due to the significant computational complexity. In particular, for all three baseline resonator topologies, first two combinations of search algorithm (AT=1,2) and three combinations of replacement type (RT=1,2,3) were considered, resulting in a total of six experiments. After this the scheme involving the L-CGA (AT=1) and replacement with the best of offspring and parents (RT=1) was determined as more preferable one. For this scheme the sensitivity analysis was performed, varying the three parameters mentioned above.

The rest of the algorithm parameters were set as follows: initial population size $NP_{max}=10$, final population size $NP_{min}=4$, number of function evaluations $NFE_{max}=500$, historical memory size H=5, initial values for memory cells $MF_{k}=0.2$, k=1,…,H, tournament size T=2. There were 5 independent runs made for every considered case. The optimization problem settings, such as dimensionality *D* and search ranges, were set depending on the resonator type, shown in Table 1: *bas* stands for baseline value, and *des* means desired value that should be reached after optimization.

Table 2, Table 3 and Table 4 contain the mean and standard deviation of best achieved values after 5 runs for the first, the second and the third case, i.e., 3-mode U-shaped resonator, 7-mode U-shaped resonator with additional cut and 5-mode W-shaped resonator. The experiments with different algorithm types and replacement types were made with η=45, PM=3 and $CR_{g}=0.6$. For cases when one of these parameters was varied, the other two were set to these values.

For the case of 3-mode resonator, the best results were achieved with both L-CGA and L-SHADGA with the first replacement scheme, i.e., the best of parents and the offspring. The worst case is the usage of L-CGA with the pairwise comparison replacement scheme. The penalty magnitude does not show any significant influence on the performance, however, the mutation and crossover distribution parameters do, in particular, smaller η values result in larger spread of sampled values and worse results. As for the crossover rate, it does not have any significant influence as well.

For the case of 6-mode resonator, the best results were achieved with L-CGA and with the second replacement scheme, i.e., random replacement with comparison. The worst case is the usage of L-SHADGA with the pairwise comparison replacement scheme. Again, the penalty magnitude does not influence the results very much. Larger η values usually result in better fitness values and the crossover rate does not have any significant influence.

For the case of 5-mode resonator, the best results were achieved with L-CGA and with the first replacement scheme, the best of parents and offspring. The worst case is again the usage of L-CGA with the pairwise comparison. The sensitivity analysis for three other parameters has similar results: larger η values are better, penalty magnitude does not show any significant influence, however, smaller $CR_{g}$ values appear to be better.

The fitness values, which are close to 1 or even smaller, are achieved usually due to small inaccuracy in $F_{0}$ frequency tuning, because the smallest possible step for $F_{0}$ is 0.5. In all cases the considered algorithms were able to find solutions that satisfied the constraints. Figure 1 and Figure 2 show the tuned topology of the 3-mode resonator, where all the parameters were found with the L-CGA algorithm using first replacement scheme.

**Figure 8:**
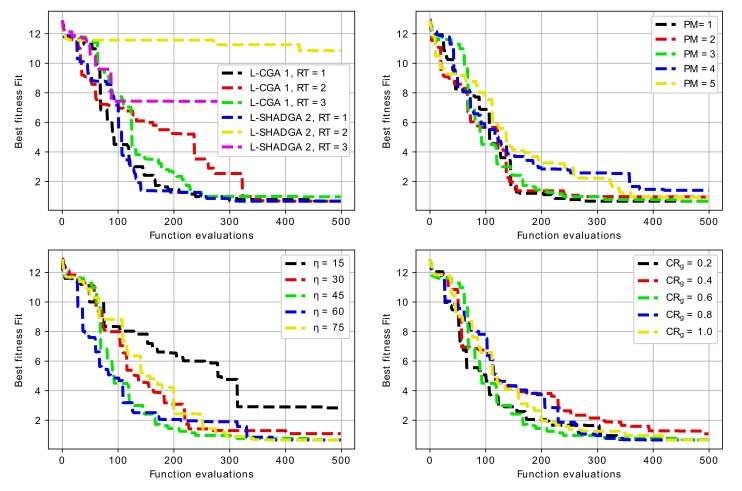
Convergence graphs of algorithms with different parameters for 3-mode resonator.

**Figure 9:**
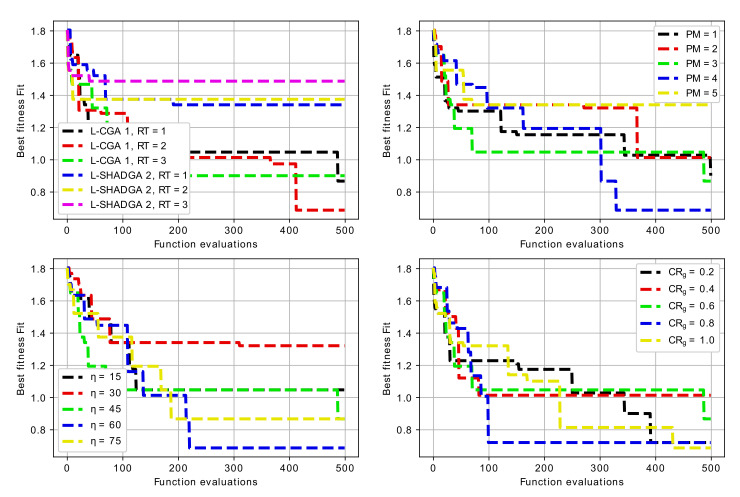
Convergence graphs of algorithms with different parameters for 6-mode resonator.

**Figure 10:**
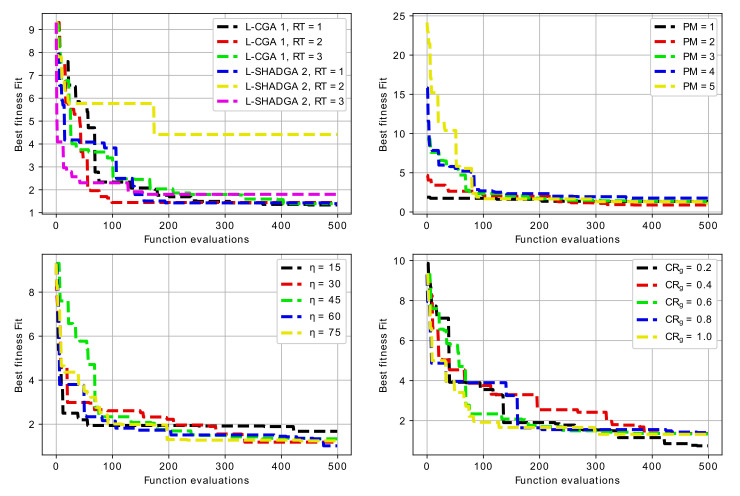
Convergence graphs of algorithms with different parameters for 5-mode resonator.

Figure 8, Figure 9 and Figure 10 contain the convergence graphs for all the performed experiments.

Figure 8, Figure 9 and Figure 10 show that all the considered algorithms were able to converge after around 300 generations in most cases. For the 3-mode resonator case, the difference between baseline and the desired frequency was larger, so the search for the optimal topology took longer. For the 6-mode and 5-mode cases the difference was not so large, and the solutions were found earlier.

In addition to the performed experiments, the optimization methods available in Matlab optimization toolbox were considered. Due to the fact that the considered problem is not smooth in terms of fitness values and cannot be differentiated, some of the methods were not able to solve such problem. The only method that was able to show competitive results was the Simulated Annealing (SA) algorithm, which was tested for all three cases with a computational resource of 500 function evaluations and recommended parameters. The same scaling of resonator size was applied to initialize the algorithm. Table 5 contains the fitness values achieved by the SA algorithm.

The SA algorithm was able to solve only the second problem, which appeared to be relatively simple, considering the convergence graphs in Figure 9. The best solutions for the first problem are significantly worse, and have much larger standard deviation, probably due to the large difference between baseline and the desired bandwidth. Comparing one of the best variants of evolutionary algorithms, L-CGA with the first replacement scheme, it should be noted that the fitness level achieved by simulated annealing (3.33) is reached after around 120 function evaluations, i.e., 25% of the total computational resource for the 3-mode resonator case. For the 6-mode case the results are comparable and reached almost with the same resource, but for 5-mode resonator case the L-CGA was able to get to the same fitness values level at around 75 generations, or 15% of the total computational resource.

For the practical use of our approach, it is much more important for the algorithm to achieve the desired values of the criteria ($F_{0}$ and dF). For other types of microwave devices which can be used as sensors, in radars, antennas and sensor network nodes, these criteria may differ. In our research, we consider $F_{0}$ and dF as examples of possible criteria. Our approach requires these criteria to be functionals of the AFC. Nevertheless, the central frequency and the width of the passband are essential for the majority of sensors and resonators regardless of their purpose. For example, for the gas sensors, the central frequency $F_{0}$ is determined by the properties of the gas to be detected. For a deeper comparison of approaches, the achieved frequency parameters are considered. In Table 6, Table 7 and Table 8 the best $F_{0}^{act}$ and $dF^{act}$ of both L-CGA with first replacement scheme and SA algorithm are given for all program runs.

For the 3-mode resonator case, both algorithms were able to get the desired $F_{0}^{act}$, however the L-CGA obtained much better $dF_{0}^{act}$ values, i.e., closer to the desired 0.7. Moreover, the standard deviation of $dF_{0}^{act}$ values was smaller.

In the 6-mode case, the average of the results received by SA is exactly 150, however, the standard deviation is larger, while L-CGA got more stable results. As for the $dF_{0}^{act}$ values, the mean and standard deviations in this case are similar, which explains similar performance in this case.

For the case of 5-mode W-shaped resonator, the average $F_{0}^{act}$ of L-CGA is not as close to the desired 150 as that of the SA, however, the standard deviation is again smaller. At the same time the $dF_{0}^{act}$ achieved by L-CGA are closer to the desired, and the standard deviations are comparable. In general, the L-CGA appears to show more stable results and is able to get to the optimal values or very close to them for both frequency and delta.

Considering these results, it can be concluded, that the developed approaches could be used for multimode resonator topology optimization when tuning a known structure to the new frequency range. The approaches presented in this study are general, and could be used for any other type of structure optimization, i.e., other resonators, filters, sensors, radars, antennas etc.

**Figure 11:**
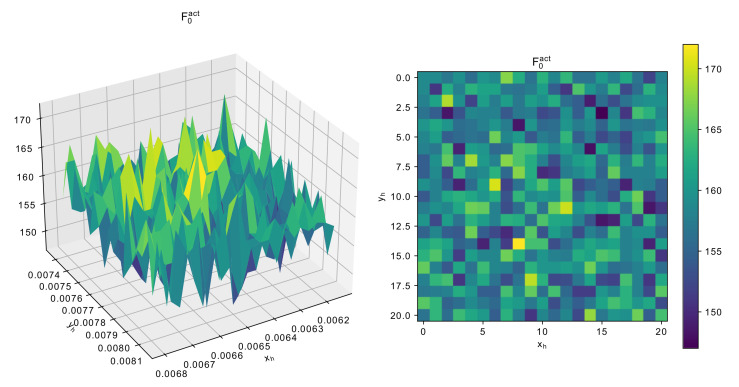
Dependence of $F_{0}^{act}$ around scaled solution for 5-mode resonator.

Although the proposed algorithms have shown to perform better than some classical approaches, it is important to consider and discuss the complexity of the optimization problems involving resonator topology optimization. To visualize the landscape of the main parameters, an additional experiment with the 5-mode resonator was performed: the two main parameters responsible for the size, namely $x_{h}$ and $y_{h}$ were varies within 5% margin from the scaled solution, i.e., the one used during initialization. Figure 11 shows the dependence of $F_{0}^{act}$ on the resonator size.

**Figure 12:**
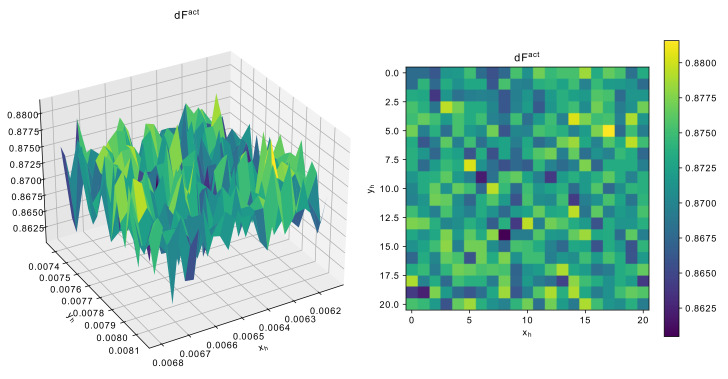
Dependence of $dF^{act}$ around scaled solution for 5-mode resonator.

**Figure 13:**
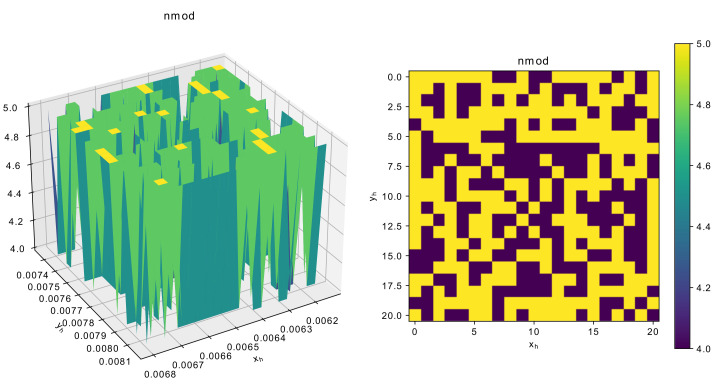
Dependence of number of modes around scaled solution for 5-mode resonator.

As can be seen from Figure 11, the landscape of the function is rather rough, that is there are many local optima, and even a small change in geometrical characteristics may cause significant change in $F_{0}$, which jumps from 150 to 170. Figure 12 and Figure 13 show similar graphs for dF and number of modes.

**Figure 14:**
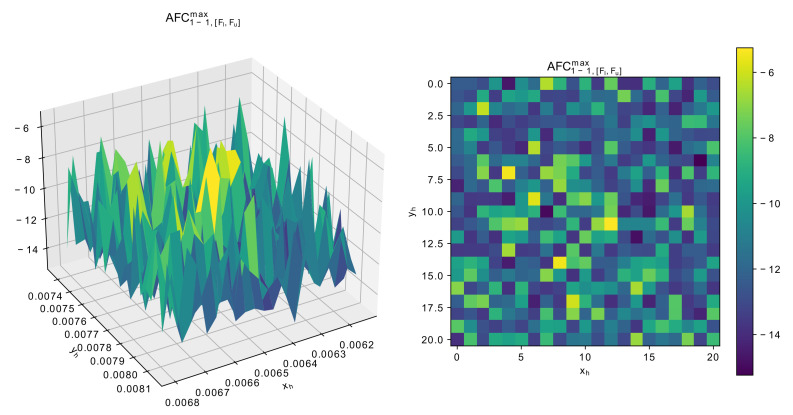
Dependence of $AFC_{1-1,[F_{l},F_{u}]}^{max}$ around scaled solution for 5-mode resonator.

Considering the landscape in Figure 12 and Figure 13, the value of dF again has multiple local optima, but the difference between worst and best parameters is not so signficiant. As for the number of modes, changing either $x_{h}$ or $y_{h}$ may cause the resonator switch to 4-mode, and it may be quite difficult to get 5 modes. Figure 14 demonstrates the $AFC_{1-1,[F_{l},F_{u}]}^{max}$ values, resposible for penalizing the solutions.

The landscape of measured $AFC_{1-1,[F_{l},F_{u}]}^{max}$ with different $x_{h}$ or $y_{h}$ around the scaled solution shows that this constraint, i.e., it should be less then −14, could be difficult to satisfy, as some of the points may get up to −6.

The analysis of landscape near the scaled solutions shows that searching for the appropriate configuration of parameter values may be difficult, as even small changes may result in very different solutions. Here, only $x_{h}$ or $y_{h}$ were considered, and the landscape analysis of other parameters, such as $x_{s1}$ or $y_{s1}$ could give some additional insights. However, considering the dimensionality and the computational complexity of the problem, it can be concluded that applying developed algorithms justified.

Our research is focused on optimization of flat resonator constructions. However, the described approach does not require the resonator structure to be planar only. The only difference is that the process of electrodynamic modelling of the 3D resonators is much more time consuming which limits the usage of our approach because of the need to run such a simulation many times. Nevertheless, the rapid growth of the computing facilities gives us a reasonable hope to use similar approach to a wider variety of shapes including 3D ones in future. The progress in the development of the 3D printing technology enables us to fabricate special microwave devices including sensors and their parts [81,82,83] for specific needs, according to individual orders. The use of biocompatible materials makes it possible to use such 3D printed devices [84] as immunosensors [85], toxicity [86] and other types of sensors. These types of sensors include both 3D spatial designs and planar designs. Our approach can be directly implemented to the planar ones.

The proposed approach to the automatic design of microwave devices includes the following stages:

Stage 1: Definition of the criteria and their desired values, and the constraints (both the criteria and left parts of the constraints must be functionals of the AFC);

Stage 2: Drawing a perspective device topology with arbitrary geometric dimensions in a specialized software tool for electrodynamic modeling;

Stage 3: Defining the variable geometric parameters to be adjusted and feasible ranges of their values;

Stage 4: Running the optimization algorithm;

Stage 5: investigation and validation of the AFC and achieved values of the criteria, return to Stage 2 if the desired values of the criteria are not achieved or the solution is infeasible;

Stage 6: Manufacturing and testing a prototype of the device.

The filters and other microwave devices used in radar sensors and sensor network nodes may be composed of several resonators [24,46]. The properties of the device depend on the topology of the resonators, number of resonators and their interconnections. Thus, for such complex cases, the optimization problem must contain both real and discrete variables. Since the DE algorithms are aimed at optimization of continuous functions, such problems may require more complex algorithms to be developed and implemented in future research. Nevertheless, the parameter adjustment of the existing topologies for the desired frequency and bandpass is an important problem, and our study greatly facilitates the solution of this problem.

The proposed algorithmic approach involves electrodynamic analysis systems of microwave device structures which is capable of simulating the amplitude-frequency response with high accuracy [12,87]. For the planar microstrip filters, electrodynamic analysis shows a good agreement of the measured and calculated characteristics of the devices (the error in the response does not exceed 3–6% (see Figure 2, Figure 4 and Figure 6) which allows us to use the results of the proposed algorithm as an accurate estimation of the characteristics of the frequency-selective properties for a device to be manufactured.

## 4. Conclusions

In this study, an important engineering problem of optimizing the topology of microstrip microwave devices was considered. To be able to solve this problem, characterized by a complex and computationally expensive fitness function and a set of non-linear constraints, we proposed two algorithms with a set of variations in algorithmic schemes and parameter values. The performed experimental comparison of the proposed algorithms on three cases, including the 3-mode U-shaped resonator, 6-mode U-shaped resonator with additional cut and 5-mode W-shaped resonator, have shown that the proposed algorithms are capable of finding optimal solutions for these topologies with relatively low computational resource. We proposed an algorithmic approach which enables us to perform automatic design of the microstrip resonators in accordance with the desired values of the critera which must be formulated as functionals of the AFC. This approach involves the electrodynamic modelling tools for estimating the AFC based on the values of the variable dimensions of the resonator topology.

We implemented our approach to three types of resonators with rather simple shapes which are nevertheless widely used in sensors, filters and radar antennas. The considered resonator types have wide bandwidth and, therefore, are promising for building selective devices with extended bandwidths, as well as sensors with an extended frequency range. However, our approach is in no way tied to the specifics of the structures we used as examples, and may be implemented for adjusting parameters of other resonator shapes as well as investigating the features of new perspective topologies

We used the center frequency $F_{0}$ and the width of the passband dF as the examples of possible criteria which may be set for the devices. Our criteria are commonly used (to obtain a narrow bandwidth commonly used in specific sensors, we must set a very low value of dF). For investigation of other perspective topologies of the resonators used as microwave sensors, parts of radar antennas or filters, new criteria may be formulated as functionals of the the AFC.

Further developments in this area may also include (but are not limited to) experimenting with self-adaptive genetic search operators, including improved local search mechanisms as well as applying surrogate models to approximate the fitness function landscape.

## Figures and Tables

**Table 1:** Algorithm parameters for three cases.

Parameter	3-Mode	6-Mode	5-Mode
$nmod^{des}$	3	6	5
*D*	5	7	7
$F_{0}^{bas}$	101.5	126	124.5
$F_{0}^{des}$	150	150	150
$dF^{bas}$	0.7	0.8	0.875
$dF^{des}$	0.7	0.8	0.875
$x_{h}^{min}$	1 mm	1 mm	1 mm
$x_{h}^{bas}$	15.5 mm	49.5 mm	78 mm
$x_{h}^{max}$	100 mm	100 mm	100 mm
$y_{h}^{min}$	1 mm	1 mm	1 mm
$y_{h}^{bas}$	280 mm	280 mm	93.5 mm
$y_{h}^{max}$	1000 mm	1000 mm	1000 mm
$x_{s1}^{min}$	0	0	0
$x_{s1}^{bas}$	0.53	0.121	0.0384
$x_{s1}^{max}$	1	1	1
$x_{s2}^{min}$	-	0	0
$x_{s2}^{bas}$	-	0.969	0.075
$x_{s2}^{max}$	-	1	0.5
$y_{s1}^{min}$	0	0	0
$y_{s1}^{bas}$	0.32	0.236	0.77
$y_{s1}^{max}$	1	1	1
$y_{s2}^{min}$	-	0	0.01
$y_{s2}^{bas}$	-	0.204	1.98
$y_{s2}^{max}$	-	1	3
$y_{p}^{min}$	0	0	0
$y_{p}^{bas}$	0.02	0.02	0.145
$y_{p}^{max}$	1	1	1

**Table 2:** Experimental results for 3-mode micrsostrip resonator.

Parameters	Mean Fitness and Standard Deviation
L-CGA, RT=1	0.67±0.00
L-SHADGA, RT=1	0.67±0.00
L-CGA, RT=2	0.96±0.59
L-SHADGA, RT=2	0.67±0.00
L-CGA, RT=3	10.86±1.55
L-SHADGA, RT=3	5.73±3.09
PM=1	0.67±0.00
PM=2	0.95±0.56
PM=3	0.67±0.00
PM=4	1.40±1.22
PM=5	0.93±0.53
η=15	2.84±1.85
η=30	1.09±0.62
η=45	0.67±0.00
η=60	0.67±0.00
η=75	0.67±0.00
$CR_{g}=0.2$	0.68±0.03
$CR_{g}=0.4$	1.08±0.60
$CR_{g}=0.6$	0.67±0.00
$CR_{g}=0.8$	0.67±0.00
$CR_{g}=1.0$	0.67±0.00

**Table 3:** Experimental results for 6-mode micrsostrip resonator.

Parameters	Mean Fitness and Standard Deviation
L-CGA, RT=1	0.87±0.52
L-SHADGA, RT=1	0.69±0.62
L-CGA, RT=2	0.90±0.00
L-SHADGA, RT=2	1.34±0.36
L-CGA, RT=3	1.38±0.39
L-SHADGA, RT=3	1.49±0.29
PM=1	0.90±0.00
PM=2	1.01±0.60
PM=3	0.87±0.52
PM=4	0.69±0.62
PM=5	1.34±0.36
η=15	1.05±0.29
η=30	1.32±0.35
η=45	0.87±0.52
η=60	0.69±0.62
η=75	0.87±0.52
$CR_{g}=0.2$	0.72±0.36
$CR_{g}=0.4$	1.01±0.60
$CR_{g}=0.6$	0.87±0.52
$CR_{g}=0.8$	0.72±0.36
$CR_{g}=1.0$	0.69±0.62

**Table 4:** Experimental results for 5-mode micrsostrip resonator.

Parameters	Mean Fitness and Standard Deviation
L-CGA, RT=1	1.34±0.77
L-SHADGA, RT=1	1.44±0.39
L-CGA, RT=2	1.39±0.28
L-SHADGA, RT=2	1.39±0.37
L-CGA, RT=3	4.42±2.10
L-SHADGA, RT=3	1.80±0.58
PM=1	1.34±0.52
PM=2	0.89±0.31
PM=3	1.34±0.77
PM=4	1.77±0.77
PM=5	1.10±0.30
η=15	1.68±0.54
η=30	1.19±0.53
η=45	1.34±0.77
η=60	1.02±0.66
η=75	1.24±0.52
$CR_{g}=0.2$	0.74±0.28
$CR_{g}=0.4$	1.36±1.32
$CR_{g}=0.6$	1.34±0.77
$CR_{g}=0.8$	1.40±0.70
$CR_{g}=1.0$	1.32±0.59

**Table 5:** Experimental results of the Simulated Annealing algorithm.

Problem	Mean Fitness and Standard Deviation
3-mode resonator	3.33±1.46
6-mode resonator	0.87±0.52
5-mode resonator	2.91±0.65

**Table 6:** Comparison of resonator characteristics achieved by L-CGA and SA, 3 mode case.

Run	L-CGA		SA	
	$F_{0}^{\mathrm{act}}$	${\mathrm{dF}}^{\mathrm{act}}$	$F_{0}^{\mathrm{act}}$	${\mathrm{dF}}^{\mathrm{act}}$
Run 1	150	0.70667	150	0.69333
Run 2	150	0.69333	150	0.66667
Run 3	150	0.69333	150	0.65333
Run 4	150	0.69333	150	0.65333
Run 5	150	0.69333	150	0.66667
Mean	150	0.696	150	0.6667
Std	0	0.005	0	0.015

**Table 7:** Comparison of resonator characteristics achieved by L-CGA and SA, 6 mode case.

Run	L-CGA		SA	
	$F_{0}^{\mathrm{act}}$	${\mathrm{dF}}^{\mathrm{act}}$	$F_{0}^{\mathrm{act}}$	${\mathrm{dF}}^{\mathrm{act}}$
Run 1	149.5	0.79599	150.5	0.80399
Run 2	148.5	0.80135	150.5	0.80399
Run 3	150	0.8	150	0.8
Run 4	149.5	0.79599	148.5	0.80135
Run 5	149.5	0.79599	150.5	0.80399
Mean	149.4	0.7978	150	0.8026
Std	0.490	0.002	0.774	0.0017

**Table 8:** Comparison of resonator characteristics achieved by L-CGA and SA, 5 mode case.

Run	L-CGA		SA	
	$F_{0}^{\mathrm{act}}$	${\mathrm{dF}}^{\mathrm{act}}$	$F_{0}^{\mathrm{act}}$	${\mathrm{dF}}^{\mathrm{act}}$
Run 1	149	0.88591	151	0.87417
Run 2	150	0.88	152	0.86842
Run 3	150	0.88	150	0.86667
Run 4	148	0.87838	149	0.87248
Run 5	149	0.87248	151	0.87417
Mean	149.2	0.8793	150.6	0.8712
Std	0.748	0.004	1.019	0.003

## Data Availability

Not applicable.

## Abbreviations

The following abbreviations are used in this manuscript:

AFC	amplitude-frequency characteristic
HPF	high-pass filter
EA	Evolutionary Algorithm
GA	Genetic Algorithm
PSO	Particle Swarm Optimization
BCH	bound constraint handling
SHA	success-history adaptation
LPSR	linear population size reduction
SBX	simulated binary crossover
